# The Fragile Site Landscape of Induced Pluripotent Stem Cells: Hierarchy, Variability, Tissue Specificity, and Links to Culture-Acquired Rearrangements

**DOI:** 10.3390/cells15171557

**Published:** 2026-08-28

**Authors:** Victoria O. Pozhitnova, Diana Zheglo, Anastasiia V. Kislova, Danila S. Kiselev, Ekaterina S. Voronina

**Affiliations:** Research Centre for Medical Genetics (RCMG), Moscow 115522, Russia; victoriapozhitnova@gmail.com (V.O.P.);

**Keywords:** induced pluripotent stem cells (IPSCs), replication stress, fragile sites, aphidicolin, caffeine, mitotic DNA synthesis (MiDAS), chromosomal aberrations

## Abstract

Induced pluripotent stem cells (iPSCs) are prone to genomic instability during prolonged culture, with recurrent chromosomal aberrations conferring selective advantages. Replication stress is a major driver of this instability, yet the repertoire of replication stress-sensitive loci in iPSCs remains largely unexplored. Here, we mapped aphidicolin-sensitive fragile sites (asFS) in three independent iPSC lines using classical cytogenetic break analysis combined with Monte Carlo simulation and MiDAS mapping directly on banded metaphase chromosomes. We identified 28 asFS, which segregated into a highly active Major cluster (8 sites, accounting for 59% of breaks among asFS) and a less active Minor cluster (20 sites). Five universal asFS (9p21, 6q25-26, 20p11-12, 10q22, Xq25) were present in all three lines, representing a fragility signature associated with the pluripotent state, with Xq25 shifting into the Major cluster after correction for X chromosome dosage. Minor asFS showed preferential co-localization with physical breakpoints or minimal overlapping regions of recurrent culture-acquired aberrations, including 20q11.21 (*BCL2L1*), 1q32 (*MDM4)*, 8q24 (*MYC*), 17q21 (*WNT3-WNT9B*), and 18q21 (*DCC/FRA18B*). MiDAS mapping validated most asFS and revealed additional replication stress-sensitive loci in pericentromeric and subtelomeric regions that are difficult to score by conventional G-banding. Comparison with fragile site maps from other cell types revealed that the iPSC asFS repertoire is distinct in rank order and relative activity, characteristic of the pluripotent state. Collectively, our findings indicate that the asFS repertoire in iPSCs is hierarchically organized into a stable universal core and a variable peripheral component, and suggest that Minor asFS may contribute to, or be associated with, the genesis of culture-acquired rearrangements. This work provides a framework for understanding how replication stress and clonal selection shape the mutational landscape of pluripotent stem cells.

## 1. Introduction

Induced pluripotent stem cells (iPSCs) hold great promise for regenerative medicine, disease modeling, and drug discovery due to their unlimited self-renewal and differentiation capacity. However, their clinical application is hampered by a high propensity for genomic instability, which emerges during reprogramming and prolonged in vitro culture. Approximately 22% of iPSC lines acquire karyotype abnormalities during cultivation [1]. These include recurrent gains of chromosomes 1q, 20q, 12, 17, and X, as well as losses of 18q, which are preferentially selected during passaging and can affect pluripotency, differentiation potential, and potentially confer tumorigenic transformation [2,3,4,5,6]. For several of these recurrent aberrations, minimal overlapping regions (MORs) have been defined, pointing to specific genomic segments that are consistently gained or lost across independent iPSC lines and often contain genes implicated in cell survival, proliferation, or pluripotency (e.g., *BCL2L1* at 20q11.21, *MYC* at 8q24, *MDM4* at 1q32, *NANOG* at 12p13.33, *TP53* at 17p13, and *SALL3* in the minimal overlapping region of 18q loss) [7]. Understanding the molecular mechanisms that drive this instability is therefore of fundamental importance.

Replication stress (RS) is inherently linked to the pluripotent cell state and is considered one of the major factors driving both numerical and structural chromosomal instability in iPSCs [1,8,9,10]. The accelerated cell cycle characteristic of pluripotency, achieved primarily by shortening the G1 phase, results in elevated basal levels of replication stress markers, including stalled replication forks, single-stranded DNA accumulation, and γH2AX phosphorylation [8,11]. Under chronic replication stress, this leads to increased rates of lagging chromosomes in anaphase, atypical mitoses, and micronuclei formation, which are well documented precursors of chromosomal rearrangements [12,13].

RS does not affect the genome uniformly. Replication-sensitive sites manifest as non-random gaps, breaks, and exchange figures on metaphase chromosomes, and include rare fragile sites (RFS), common fragile sites (CFSs), early-replicating fragile sites (ERFSs) [14], as well as fragile centromeres [15] and telomeres [16]. At the molecular level, these regions are intrinsically difficult to replicate due to repetitive sequences, secondary structures, transcription-replication collisions, and large replicons that delay fork progression. Consequently, such loci are prone to entering mitosis with under-replicated DNA. This gives rise to ultrafine anaphase bridges, which can lead to chromosome missegregation and segmental loss [17]. To resolve these structures, cells engage mitotic DNA synthesis (MiDAS), which is initiated by the structure-specific nuclease MUS81-EME1 and proceeds through RAD52-mediated strand annealing and POLD3-dependent synthesis [18,19].

The repertoire of RS-sensitive fragile sites is cell-type specific and shows inter-individual variability [20,21]. Historically, such sites have been mapped in lymphocytes and fibroblasts using classical cytogenetics [22,23]. Only a limited number of studies have addressed other histological origins [20,24,25], and the landscape of RS-sensitive sites in pluripotent stem cells remains largely unexplored [26]. Later, alternative markers of RS-sensitive regions have been applied for high-resolution global mapping of fragile sites, such as aphidicolin-induced FANCD2 ChIP-seq [27,28], Repli-seq [29,30] and MiDAS-seq [31], which showed high concordance with sites of chromosome fragility. Recently, the ERFS repertoire in embryonic pluripotent stem cells was mapped by colocalization of γH2AX, RPA, BRCA1, and SMC5 in early replicating regions under HU-induced stress [32]. Many of these loci fall within promoters and enhancers of genes controlling pluripotency, proliferation, and genome integrity, and overlap with cancer-associated CNVs and SNVs.

In this study, we combined classical cytogenetic break mapping with Monte Carlo simulation to identify loci of non-random breakage in three independent iPSC lines exposed to aphidicolin-induced RS. To functionally validate the identified loci, we performed MiDAS mapping directly on banded metaphase chromosomes, which detects RS-induced repair sites at cytogenetic resolution and is less dependent on optimal chromosome morphology. Finally, we compared the obtained map of aphidicolin-sensitive fragile sites (asFS) with breakpoints of recurrent chromosomal rearrangements that accumulate during long-term iPSC culture.

Our results reveal that the asFS repertoire in iPSCs is hierarchical, consisting of highly active (Major) and moderately active (Minor) clusters. Notably, several Minor asFS co-localize with breakpoints of recurrent aberrations, suggesting their involvement in adaptive, culture-acquired rearrangements. Together, our findings indicate that the interplay between replication stress and subsequent clonal selection shapes the mutational landscape of iPSCs, providing a framework for improving culture conditions and monitoring genetic stability in pluripotent stem cells.

## 2. Materials and Methods

### 2.1. Cell Lines and Culture Conditions

Three human induced pluripotent stem cell lines derived by skin fibroblast reprogramming using non-integrating vectors, namely, P1L5 (hPSCreg name RCMGi001-A, RRID: CVCL_YT36; BioSamples ID: SAMEA114313271) [33], P12L3 (hPSCreg name RCMGi017-A, RRID: CVCL_C9HG; BioSamples ID: SAMEA115128016), and P16L1 (hPSCreg name RCMGi015-A, RRID: CVCL_E4EN; BioSamples ID: SAMEA115108458), were obtained from the Biobank “All-Russian Collection of Biological Samples of Hereditary Diseases” (Research Centre for Medical Genetics, Moscow, Russia). These cells were cultured on Vitronectin-coated plates (A14700, Gibco™, Hong Kong, China) in Essential 8™ (A1517001, Gibco™) or Gibris 8 (C780E, Pan-eco-ltd, Moscow, Russia) medium supplemented with 50 U/mL penicillin and 50 μg/mL streptomycin. Treatments were performed when cells reached approximately 50–60% confluency, at least two days after reseeding. Replication stress was induced by adding aphidicolin (A4487, Sigma Aldrich, Saint Louis, MO, USA) at a final concentration of 0.1μM, together with 1 mM caffeine (C0750, ReagentPlus^®^, Darmstadt, Germany), 21 h before cell harvest. For MiDAS detection, 20 μM EdU was added 40 min prior to cell harvest. MiDAS analysis was performed in separate experiments on separate slides.

### 2.2. Metaphase Spread Preparation and Chromosome Banding

For metaphase preparation, cells were synchronized with 0.1 μg/mL Demecolcine (D7385, Sigma Aldrich, Saint Louis, MO, USA) for 1 h, harvested by trypsinization (Pan-eco-ltd, Russia), hypotonized in prewarmed 0.55% KCl for 13–14 min, and fixed in three changes of ice-cold methanol/glacial acetic acid (3:1, Chimmed, Moscow, Russia). The cell suspension was dropped onto microscope glass slides (Menzel Glaser, Braunschweig, Germany) and then air-dried. To obtain G-like banded chromosomes, slides were stained with Vectashield anti-fade mounting medium with DAPI (H-1200, Vector Labs, Newark, CA, USA) and premixed 0.3 mg/mL Actinomycin D (1071001, Serva, Heidelberg, Germany) for band contrast. Metaphase images were acquired using an AxioImager 2 (Zeiss, Oberkochen, Germany) microscope and processed using the Q-banding mode of Case Data Manager 6.0 software (Applied Spectral Imaging, Carlsbad, CA, USA). Chromosomal break locations were analyzed according to the International System for Human Cytogenomic Nomenclature (ISCN 2024) [34].

### 2.3. MiDAS Detection

EdU incorporation was detected using the Click-iT™ EdU Cell Proliferation Kit for Imaging (C10339, Invitrogen, Carlsbad, CA, USA) according to the manufacturer’s instructions. Briefly, slides were blocked in 1% BSA, permeabilized with 0.5% Triton X-100 in PBS, incubated with Alexa-594-picolyl azide in the supplied click buffer, and then washed three times with PBS/Triton. Slides were counterstained with Glycerol Mounting Medium with DAPI (ab188804, Abcam, Waltham, MA, USA). For MiDAS mapping, we analyzed 62 metaphases for P1L5 (55 with breaks and 7 normal), 29 for P12L3, and 221 for P16L1 (85 with EdU signals), scoring a total of 451 EdU-positive signals across the three lines (147 in P1L5, 153 in P12L3, and 151 in P16L1).

### 2.4. Monte Carlo Simulation for Threshold Determination

To distinguish genuine fragile sites from randomly distributed breaks, we performed Monte Carlo simulations under the null hypothesis that breaks are uniformly distributed across all cytogenetic bands. The human haploid karyotype contains approximately 400 bands at standard resolution (ISCN 2024); each band was treated as a single analytical unit. For each simulation, the total number of breaks observed (N) was randomly assigned to the 400 bands with equal probability (*p* = 1/400), following a multinomial distribution. In each of 100,000 iterations, the maximum number of breaks occurring in any single band was recorded. The 99th percentile of this distribution was chosen as the significance threshold, corresponding to a one-tailed false positive rate of 1%. For the pooled dataset (966 breaks), the threshold was 11 breaks per locus. For individual lines, the thresholds were 7 breaks for P1L5 (342 breaks), and 6 breaks for both P12L3 (318 breaks) and P16L1 (306 breaks). The 95th percentiles were also computed for comparison (pooled: 9 breaks; per line: 5 breaks) and are provided in Supplementary Table S1. Simulations were performed in Python 3.12 using NumPy 2.0.2 with a fixed random seed (42) for reproducibility.

### 2.5. Identification and Classification of asFS

For break analysis, we scored 173 metaphases for P1L5, 133 for P12L3, and 267 for P16L1, yielding 342, 318, and 306 chromatid breaks, respectively. Loci with 11 or more breaks across all three lines were designated as significant aphidicolin-sensitive fragile sites (asFS). Each asFS was then examined for its activity pattern across individual lines using per-line thresholds (≥7 for P1L5, ≥6 for P12L3, and ≥6 for P16L1). Based on these patterns, loci were classified as universal (significant in all three lines), repressed (significant in two lines), line-specific (significant in only one line), or pool-only (significant only by pooled threshold). The classification is summarized in Supplementary Table S1.

### 2.6. Hierarchical Clustering by Break Frequency

To identify intrinsic groupings among the 28 significant asFS based on break count distribution, we performed k-means clustering validated by the gap statistic and the elbow method. Both approaches consistently supported a two-cluster solution, which was further reinforced by a natural gap of 9 breaks between the 7th and 8th loci (34 vs. 25 breaks). After correction for X chromosome dosage in the male line (P1L5), Xq25 shifted into the first cluster, resulting in a Major cluster of 8 loci and a Minor cluster of 20 loci. Details are provided in Supplementary Figure S2 and Supplementary Table S1.

## 3. Results

### 3.1. The Landscape of Aphidicolin-Sensitive Fragile Sites in iPSCs

To induce chromatid breaks, we treated cells with aphidicolin, a reversible inhibitor of replicative polymerases, combined with caffeine to attenuate ATR and ATM signaling and thereby override cell-cycle arrest and cell death. Metaphase chromosomes were harvested 21 h after treatment initiation. This duration has been shown to permit completion of a full S-phase under replication stress conditions [35]. The experimental conditions, therefore, allow for the induction of both ERFSs [13] and canonical CFSs. Across three independent iPSC lines, we scored 966 chromatid breaks distributed over 110 distinct cytogenetic bands (Supplementary Table S1).

The traditional threshold for fragile site identification, empirically established at ≥1% of total breaks when scoring approximately 300 breaks, has been widely used to define non-randomly fragile loci [20]. In this study, to distinguish genuine asFS from random background breakage, we performed Monte Carlo simulations of random break distribution across the 400 bands resolvable at standard cytogenetic resolution. At the 99% confidence level, the pooled threshold for non-random break accumulation was 11 breaks per locus across all three lines (Supplementary Figure S1). This threshold is equivalent to 1.2% of breaks and thus slightly exceeds the traditionally used cutoff. For individual lines, the thresholds were 7 breaks for P1L5 and 6 breaks for P12L3 and P16L1, reflecting the higher stringency required to call a locus significantly fragile in a single line.

**Figure 1:**
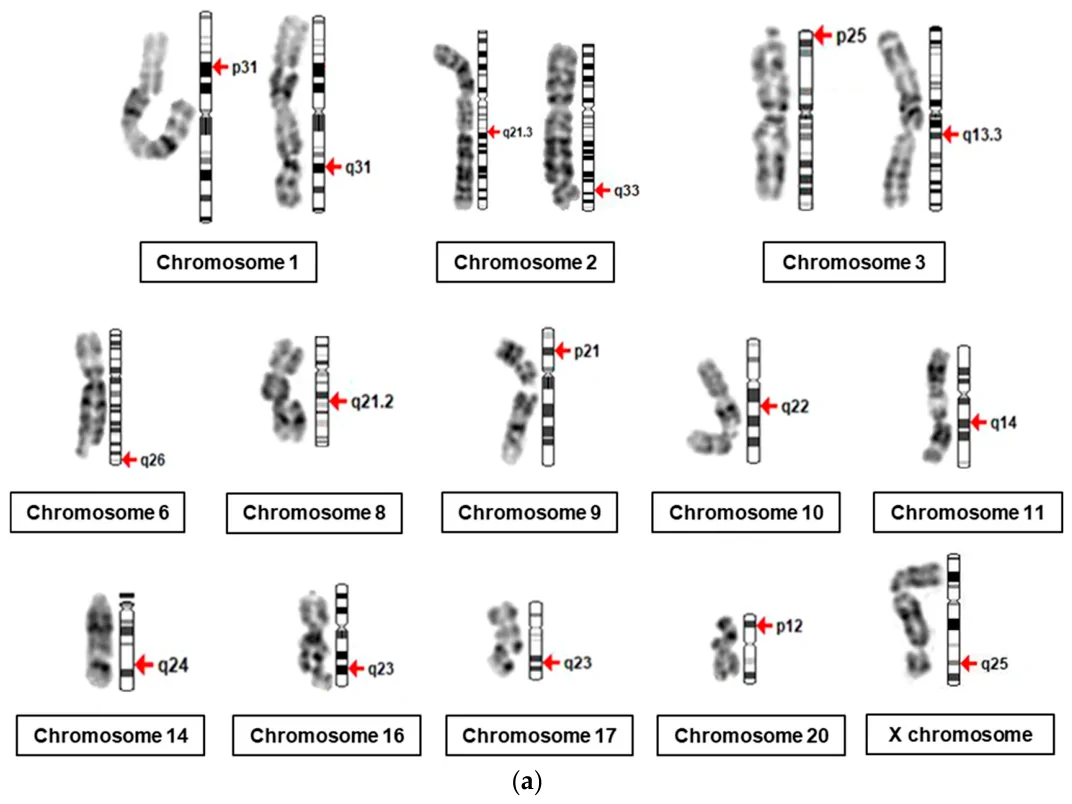

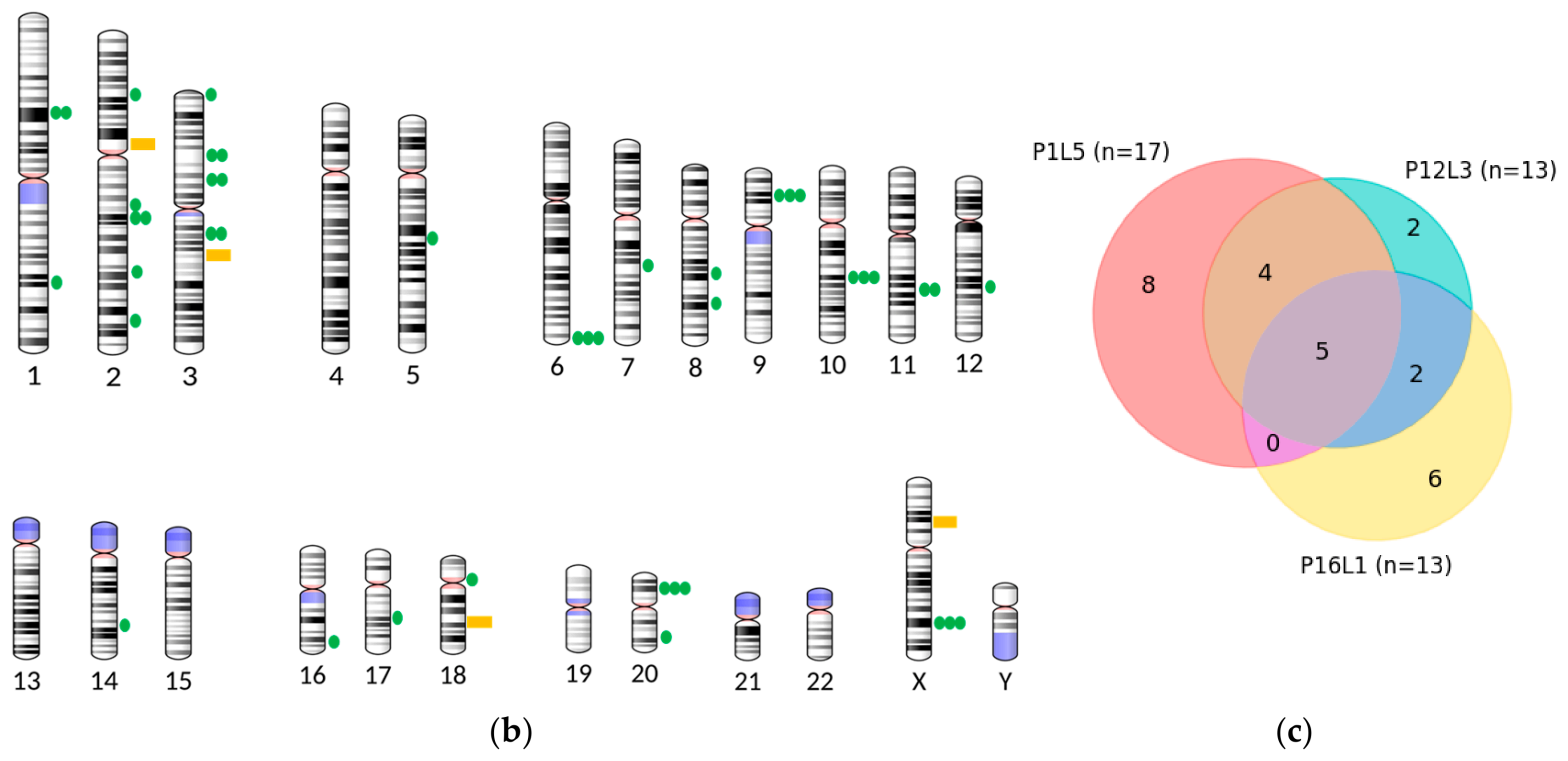
Mapping of aphidicolin-sensitive fragile sites (asFS) in iPSCs. (**a**) Representative images of chromatid breaks on banded metaphase chromosomes at the most active asFS loci. Arrows indicate breaks at the indicated chromosomal bands; (**b**) ideogram of all 31 asFS mapped to human chromosomes. Each green dot represents an asFS locus; the number of dots per locus corresponds to the number of iPSC lines (P1L5, P12L3, P16L1) in which the locus is significantly fragile according to the Monte Carlo test (≥5 breaks per line). Orange boxes indicate loci that are significant only by the pooled threshold (≥9 breaks total) but do not reach significance in any individual line. Purple indicates polymorphic regions of human chromosomes and pink indicates centromeres. (**c**) Venn diagram of loci exceeding per-line significance thresholds across three iPSC lines. Each circle represents the set of loci significant in P1L5 (red), P12L3 (teal), or P16L1 (yellow). Numbers indicate loci in each intersection. The central intersection of five loci (9p21, 6q25-26, 20p11-12, 10q22, and Xq25) represents the universal fragility signature.

Applying the pooled threshold (≥11 breaks), we identified 28 loci with significant non-random break accumulation (Figure 1a,b). These 28 asFS accounted for 691 of 966 breaks (71.6%), demonstrating that they represent the principal targets of aphidicolin-induced chromosomal damage in iPSCs. The remaining 305 breaks (28.4%) were distributed across other 82 bands at frequencies below the statistical threshold, consistent with either stochastic breakage or low activity asFS that fall below the detection limit of this analysis.

### 3.2. Conservation of the Fragility Pattern Across Lines

The activity of the 28 asFS identified in the pooled analysis varied considerably among the three lines. When we applied the more stringent per-line thresholds, 17 loci reached significance in P1L5, 13 in P12L3, and 13 in P16L1 (Figure 1b,c, Supplementary Table S1). These inter-line differences allowed us to classify the 28 asFS into four categories based on their conservation across lines.

Five loci exceeded the per-line threshold in all three lines: 9p21, 6q25-26, 20p11-12, 10q22, and Xq25. These universal asFS are constitutively fragile and represent the minimal fragility signature of the iPSC state. Six loci showed a repressed pattern, being active in two lines but significantly suppressed in the third. Repression occurred predominantly in P16L1 (four loci), with two additional loci repressed in P1L5, while P12L3 showed no repressed loci. This asymmetric distribution suggests that P16L1 has a distinct epigenetic landscape or a higher propensity for context-dependent modulation. The remaining loci comprised line-specific (13 loci) and pool-only (4 loci) sites. In addition, three loci that did not meet the pooled threshold were significant in individual lines (8q21-22 and 18p11.2 in P16L1, and 8q23-24 in P1L5), indicating that the full repertoire of aphidicolin-sensitive fragility in iPSCs extends beyond the 28 loci captured by pooled analysis.

Inter-individual variability of fragile site landscapes has been previously observed in primary lymphocytes and dermal fibroblasts, with the level of conservation estimated at 45–48% [20], which is comparable to our findings. The discrepancies between the pooled set and individual lines may arise from genuine donor-specific epigenetic variation, microdeletions within a fragile site, or stochastic sampling due to the finite number of metaphases examined. Consequently, the absence of a locus from an individual line’s significant list does not necessarily imply biological absence, as it may be active but at a level below our detection limit.

### 3.3. Major and Minor asFS: Two Tiers of Genomic Instability

**Figure 2:**
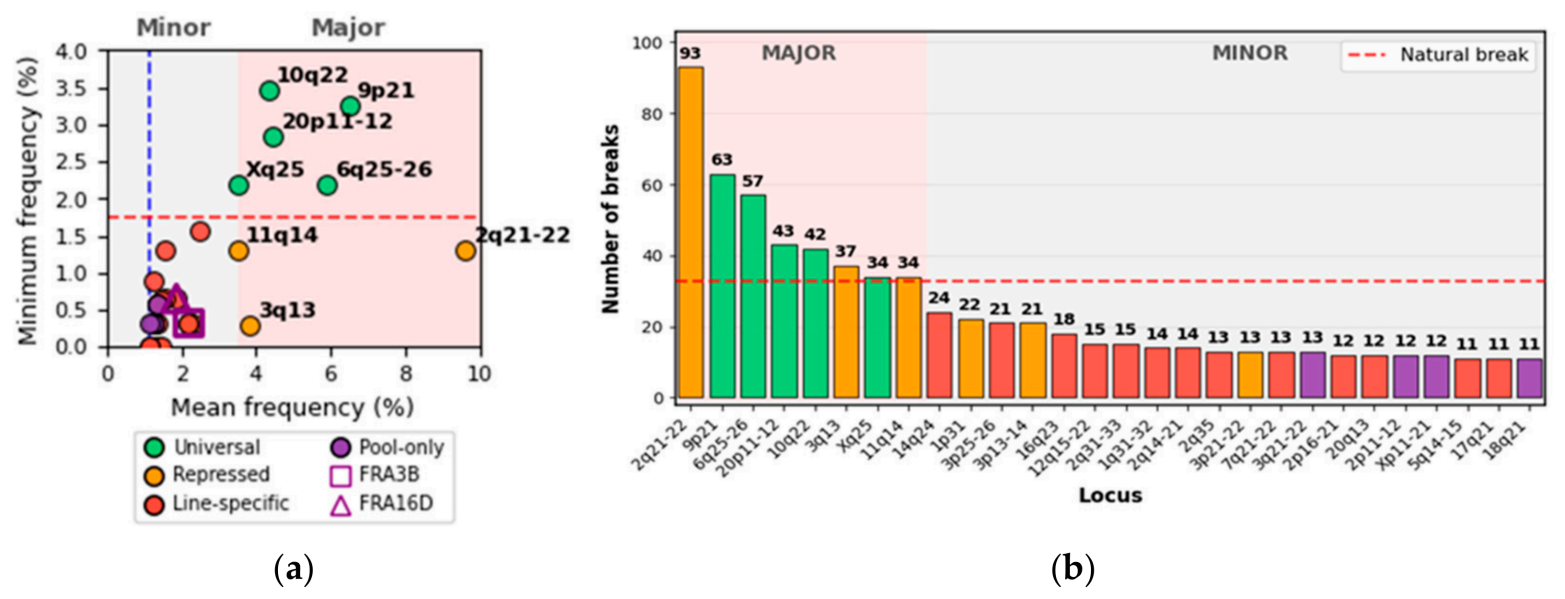
Break count distribution across 28 asFS in iPSCs. (**a**) Scatter plot of cytogenetic break distribution across 28 asFS in iPSCs. Each point represents a cytogenetic locus, plotted by its mean break frequency (x axis) versus its minimum frequency across the three lines (y axis). Loci are color-coded according to their conservation pattern: universal (green), repressed (orange), line-specific (red), pool-only (purple). The dashed vertical line indicates the pooled Monte Carlo threshold (1.14%, equivalent to 11 breaks). The dashed horizontal line marks the per-line significance threshold (1.75%, equivalent to 7 breaks in P1L5), below which a locus fails to reach significance in at least one individual line. Background shading separates the Major cluster (pink) from the Minor cluster (gray). FRA3B and FRA16D are highlighted. Universal loci and Major cluster repressed loci are labeled with band names. (**b**) Loci are ranked by total break count across three lines. Bars are color coded according to conservation pattern: universal (green), repressed (orange), line-specific (red), pool-only (purple). Background shading separates the Major cluster (pink) from the Minor cluster (gray), with the boundary determined by k-means clustering. The dashed red line marks the natural break between clusters at 33 breaks. Numbers above bars indicate break counts in the pooled dataset.

When we examined the distribution of break counts among the 28 asFS, a striking hierarchical structure emerged. K-means clustering, validated by silhouette analysis and the gap statistic, consistently resolved the loci into two distinct groups separated by a natural gap between the 7th and 8th loci (34 vs. 25 breaks) (Supplementary Figure S2a). To account for the single X chromosome copy in the male line (P1L5), we corrected the break count of Xq25 by doubling its contribution from this line. This adjustment yielded a total of 34 breaks for Xq25, shifting it into the Major cluster and bringing all five universal loci into the top tier (Figure 2a,b). The Major cluster thus comprised eight loci with break counts ranging from 34 to 93 (mean 49.4), accounting for 59% of all breaks occurring within asFS, despite representing only 29% of the loci. These eight loci were 2q21-22, 9p21, 6q25-26, 20p11-12, 10q22, 3q13, 11q14, and Xq25. The Minor cluster included the remaining 20 loci with break counts ranging from 11 to 25 (mean 15.6), accounting for the remaining 41% of breaks. This group contained all repressed, line-specific, and pool-only loci, reflecting their more variable and context-dependent expression. If X inactivation in female lines additionally reduces fragile site expression, the true activity of Xq25 on the active X homolog would be even higher than estimated here.

Our observation is in line with previous reports indicating that a relatively small number of fragile loci account for the majority of chromosomal breaks under replication stress conditions [20]. The Major cluster thus represents a small set of dominant fragility hotspots that collectively account for the majority of replication stress-induced chromosomal damage in iPSCs. Their universal conservation across independent lines suggests they are intrinsic to the pluripotent state. Conversely, the Minor cluster comprises a larger and more heterogeneous group of sites with lower baseline fragility that are more susceptible to line-specific modulation, whether by epigenetic differences, genetic background, or stochastic variation during reprogramming.

### 3.4. MiDAS Mapping Refines the Landscape of Aphidicolin-Sensitive Loci

**Figure 3:**
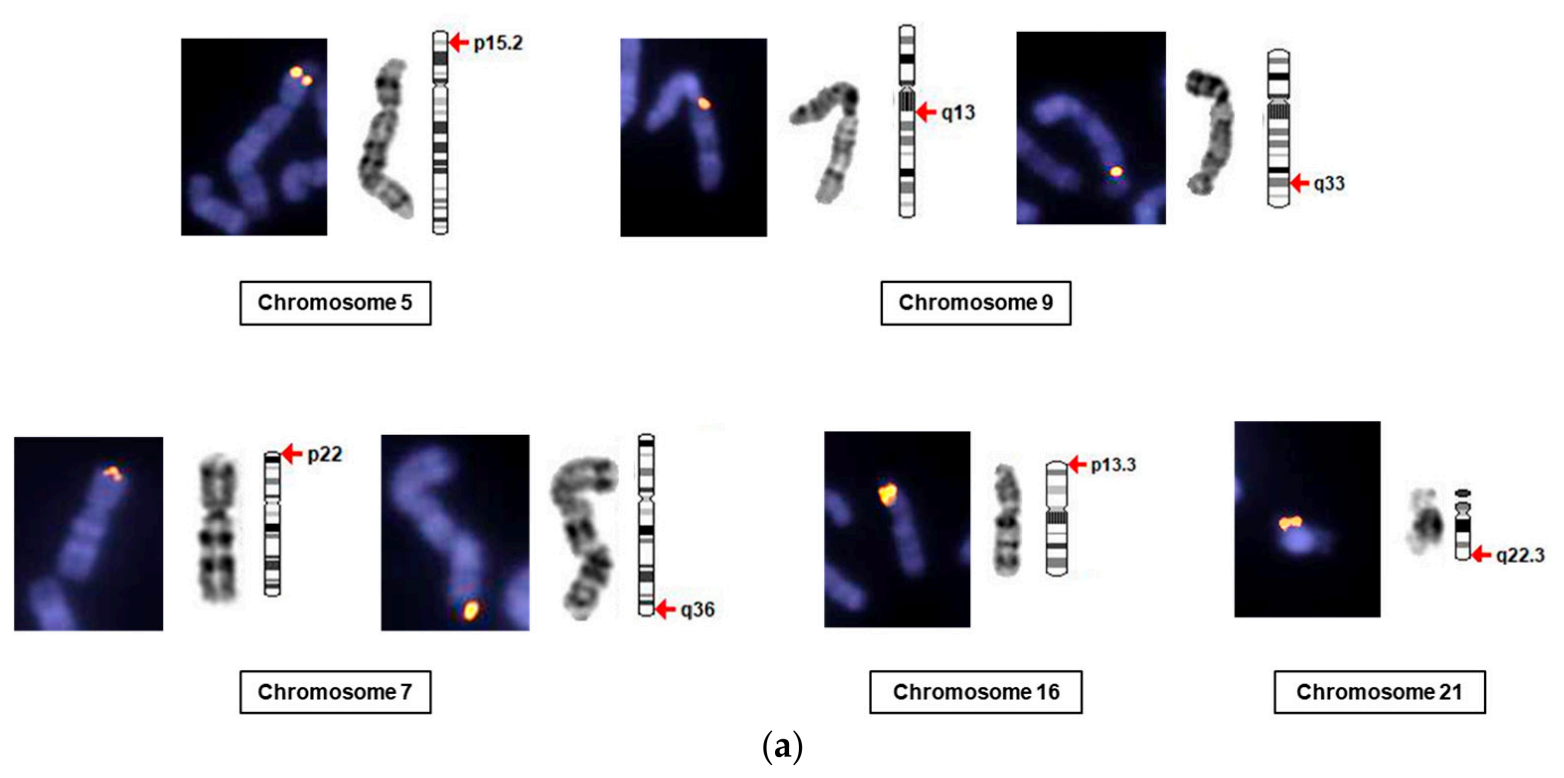

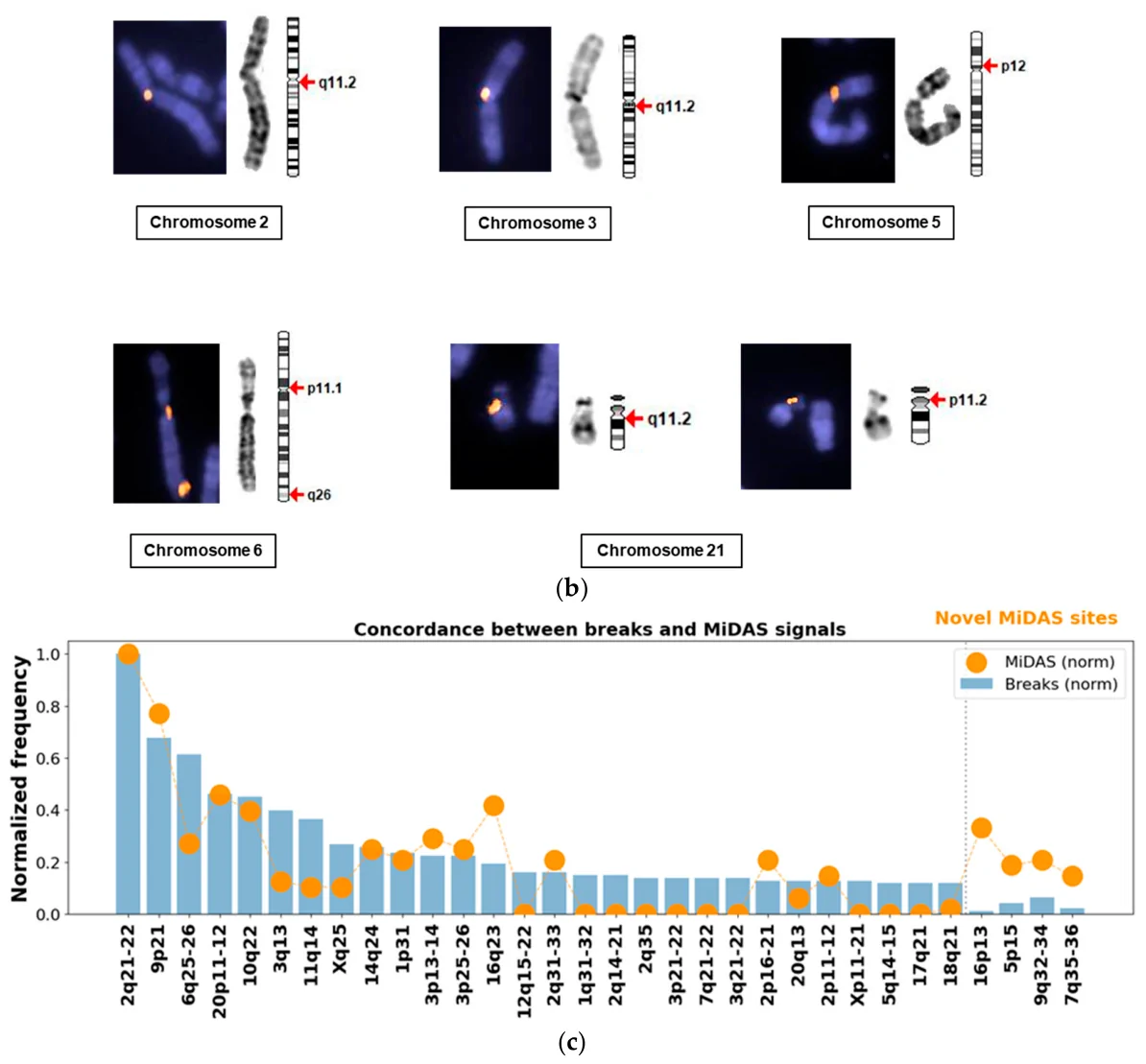
Cytogenetic mapping of aphidicolin-induced MiDAS sites in iPSCs: (**a**) representative images of MiDAS signals at cytogenetically challenging regions and newly identified RS-sensitive loci; (**b**) centromeric MiDAS signals; (**c**) concordance between chromatid break frequencies and MiDAS signals across aphidicolin-sensitive fragile sites (asFS) in iPSCs. Loci are ranked from left to right by decreasing total break counts. Blue bars represent normalized break frequencies; orange circles indicate normalized MiDAS signal counts. The dashed orange line connects MiDAS values to highlight deviations from the break-based ranking. Normalization was performed relative to the maximum value within each dataset to allow direct comparison of the two profiles. The overall ranking is largely consistent between the two methods, with most loci showing proportional break and MiDAS signals. Notable discrepancies are observed for 16p13, 5p15, 9q32-34, 7q35-36, which show higher MiDAS signals than expected from break counts, and for 3q13 and 11q14, which show lower MiDAS signals despite high break frequencies. Four loci not identified as asFS by break counting, 16p13, 5p15, 9q32-34 and 7q35-36, are shown separately on the right side of the plot as novel MiDAS-positive candidates with high signal counts.

To independently validate the 28 asFS identified by break counting and to capture loci potentially missed in cytogenetically challenging regions, we mapped MiDAS sites in the same three iPSC lines. Following 21 h aphidicolin/caffeine treatment and synchronization at the G2/M boundary, cells were released into mitosis and pulse-labeled with EdU to detect DNA synthesis. A total of 451 MiDAS signals were scored on metaphase chromosome spreads (147 in P1L5, 153 in P12L3, and 151 in P16L1) and mapped to cytogenetic bands (Figure 3a,b, Supplementary Table S2).

We observed concordance between MiDAS- and break-based asFS for multiple loci (Figure 3c). For several of them, the interline distribution of MiDAS signals closely paralleled that of chromatid breaks, most notably at 2q21-22, where both methods showed the highest activity in P12L3, intermediate activity in P1L5, and the lowest activity in P16L1. This agreement across independent methods suggests that both report genuine biological fragility rather than method-specific artifacts. Of the 28 asFS, 22 (79%) showed MiDAS signals in at least one line. All eight Major cluster loci exhibited robust MiDAS signals across all three lines, confirming their status as genuine fragility hotspots. The six asFS without detectable MiDAS signals were exclusively Minor cluster loci with lower break counts, which may reflect technical limitations of the EdU pulse-labeling window or the relatively small number of MiDAS events scored per line, rather than the absence of mitotic DNA synthesis at these sites.

Comparison of the activity clusters between chromosomal breaks and MiDAS signals revealed a partial but not absolute concordance. While most Major asFS showed strong MiDAS signals, two (3q13 and 11q14) exhibited unexpectedly low MiDAS counts (six and five signals, respectively), despite their high break frequencies. Xq25, which shifts to the Major cluster after X chromosome dosage correction in the male line, showed moderate MiDAS signals (7 signals), consistent with its position among the lower-activity Major sites. Conversely, several Minor asFS displayed MiDAS signals comparable to or even exceeding those of Major sites. Notably, 16q23, a Minor cluster locus by break count, showed prominent MiDAS signals across all three lines (Figure 3c, Supplementary Table S2).

Beyond confirming known asFS, MiDAS mapping revealed novel MiDAS-positive loci that had not previously been identified by break counting. These include 16p13, 5p15, 9q32-34, and 7q35-36, all of which exhibited MiDAS signals comparable to those of Major sites despite having few or no breaks (Figure 3a,c). These loci map to cytogenetically challenging regions (pericentromeric, subtelomeric, and pale G-bands), where chromatid breaks are difficult to score by conventional G-banding. These results highlight the complementary nature of both approaches: break counting identifies direct chromosomal damage, while MiDAS captures repair-associated events and can reveal fragility in regions that are systematically underrepresented in cytogenetic analysis. Together, they provide a more complete picture of the aphidicolin-sensitive landscape in iPSCs.

### 3.5. Tissue-Specific Fragile Site Landscape in iPSCs

Comparison with published fragile site maps from somatic cells [20,23] reveals that while some loci are shared across cell types, their relative activities differ substantially. For example, *FRA3B* and *FRA16D*, which rank among the most active fragile sites in lymphocytes and fibroblasts, fall into the Minor cluster in iPSCs, whereas FRA2Ftel emerges as the most active locus in iPSCs but is less prominent in somatic cells. This remodeling of the fragile site hierarchy upon reprogramming is consistent with previous reports of cell-type-dependent fragility [20,23,36]. Furthermore, the observation by Mrasek et al. [23] that aphidicolin induces a wide range of both rare and common fragile sites in lymphocytes supports our use of the term ‘aphidicolin-sensitive’ rather than ‘aphidicolin-specific’, as it highlights the broad vulnerability of these loci to replication stress rather than their exclusivity to a single inducing agent. This comparison supports the notion that the fragile site repertoire is remodeled upon reprogramming.

Comparison of the iPSC asFS repertoire with canonical fragile sites described in somatic cell types reveals substantial tissue-specific differences. Two of the most extensively characterized common fragile sites in human lymphocytes and fibroblasts, *FRA3B* (3p13-14) and *FRA16D* (16q23), were both identified within our iPSC dataset, but neither dominated the landscape. *FRA3B* (21 breaks) fell into the Minor cluster and showed repression in P16L1, while *FRA16D* (18 breaks) was line-specific to P1L5. Their relatively modest activity in iPSCs contrasts sharply with their well-documented prominence in lymphocytes, fibroblasts and epithelial cells [20,23], where they consistently rank among the most frequently expressed fragile sites. This reduced activity supports the notion that the fragile site landscape undergoes substantial remodeling upon reprogramming to pluripotency.

Conversely, *FRA2Ftel* (2q21-22), a fragile site that has received less attention in somatic cell studies, emerged as the most active locus in our iPSC dataset (93 breaks), exhibiting pronounced line-specific differences (highest in P12L3, intermediate in P1L5, lowest in P16L1). Its high activity across all three lines suggests that certain loci may acquire or maintain fragility in the pluripotent state, possibly reflecting the altered replication program and chromatin landscape characteristic of iPSCs.

Comparison with lymphocytes, fibroblasts, and epithelial cells shows that the iPSC asFS repertoire is distinct in rank order and relative activity, although some loci are retained across cell types [20,25]. This tissue-specific remodeling suggests that the regions most vulnerable to replication stress differ fundamentally between pluripotent and differentiated cells, reflecting the unique biology of the pluripotent state. Throughout this section, we use the term ‘tissue-specific’ in its broad sense, encompassing differences between cell types at various stages of differentiation. The observed remodeling of the fragile site landscape likely reflects both the acquisition of pluripotency and the altered replication program characteristic of iPSCs. Future studies comparing iPSCs with their somatic cell of origin and with embryonic stem cells will be required to dissect these contributions.

### 3.6. Co-Localization of Minor asFS with Recurrent Aberration Breakpoints

To assess whether the identified asFS may contribute to culture-acquired chromosomal rearrangements, we compared their genomic localization with known physical breakpoints and minimal overlapping regions (MORs) of recurrent aberrations documented in long-term iPSC cultures. The universal Major asFS (9p21, 6q25-26, 20p11-12, Xq25, 14q24) does not coincide with any known recurrent rearrangement hotspot.

### 3.7. 1q Gain

Interstitial duplications of 1q or derivative chromosomes with various translocation partners are among the most frequent structural aberrations recurrently arising in cultured iPSCs and their derivatives. The minimal overlapping region (MOR) encompasses the *MDM4* oncogene at 1q32. Varying breakpoints have been reported, including centromeric regions, 1q21, 1q23, 1q25, and other bands [1,37,38]. At the molecular level, SNP array analysis mapped breakpoints of two independent aberrations to q23.3–q42.12 and q21.2 [39]. In our study, two Minor asFS were identified on chromosome 1: at 1p31 (rank 10) and 1q31-32 (rank 16). The latter falls within the MOR of recurrent 1q gain. In addition, several non-significant breakage sites (1q42, 6 breaks; 1q24-25, 3 breaks) and MiDAS-positive loci (1q22-31, 4 signals; 1q12, 1 signal; 1q32, 1 signal) were detected, suggesting that this region may harbor additional low-level fragility.

### 3.8. Amplification of 20q11.21

Recurrent gain of the 20q11.21 region occurs through tandem duplications, triplications, isochromosome formation, and other gross rearrangements with varying breakpoints [40,41,42]. The MOR of 20q gain centers on *BCL2L1*, an anti-apoptotic gene that confers a selective growth advantage. In our study, a fragile and MiDAS-positive band was identified at 20q13 (*FRA20E*), which lies distal to the core 20q11.21 amplification region. Although 20q13 does not overlap the main MOR, its involvement in the formation of 20q amplifications cannot be excluded, as breakpoints may extend to the distal q-arm in some rearrangements.

### 3.9. 18q Loss

Loss of 18q is a recurrent event in cultured iPSCs, frequently associated with reduced differentiation potential. The breakpoint has been mapped within the *DCC* gene at 18q21.2 [5,39], whereas the MOR contains *SALL3*, a gene implicated in pluripotency regulation. In our dataset, we identified *FRA18B* at 18q21 as a Minor asFS (18q21, rank 27), which directly overlaps the physical breakpoint within *DCC*. This suggests that the fragility at *FRA18B* may contribute to the initiation of 18q deletions.

### 3.10. 8q Gain

Gain of 8q, particularly at the *MYC* locus (8q24), is a well-documented recurrent aberration in iPSCs. The MOR centers on *MYC*, a key oncogene driving proliferation. Although physical breakpoints for 8q gain have not been precisely mapped, we identified a Minor asFS at 8q23-24 (*FRA8C/D*), which lies within the MOR of 8q gain. This localization raises the possibility that fragility at this site may contribute to 8q amplification events, though further molecular characterization is needed.

### 3.11. 12p Gain

Amplification of 12p13.33, encompassing the pluripotency factor *NANOG*, is a common adaptive change in iPSC cultures [43]. No asFS were identified on the short arm of chromosome 12; the only asFS on this chromosome is located on the opposite arm at 12q15-22. Of note, a fragile site at 12q23.1 (*FRA12L*), spanning the large neuronal gene *ANKS1B*, has recently been mapped in iPSCs and shown to contribute to chromosome 12 missegregation and rearrangements [35].

### 3.12. 17q Gain, 17p Loss

The short arm of chromosome 17, particularly the 17p13.1 region containing the *TP53* gene, is a site of sporadic deletions that can confer a selective growth advantage. Additionally, the 17q21.31/*WNT3-WNT9B* amplification has been reported as a “hot spot” for genetic variation altering cell phenotype [44]. In our study, two fragile loci were identified on chromosome 17. The locus 17q21 (11 breaks) exceeded the pooled significance threshold, while 17q22-23 (6 breaks) did not.

Overall, our findings support a model in which the recurrent aberration landscape of cultured iPSCs arises from the interplay between replication-stress-induced fragility and subsequent clonal selection for growth-promoting changes. Minor asFS and even non-significant break sites appear to be preferentially captured in culture-acquired rearrangements, whereas the most active Major loci are conspicuously absent from known recurrent breakpoints. This suggests that regions of extreme fragility may produce lesions that are either lethal or lack selective advantage, whereas moderately fragile sites provide viable substrates for clonal expansion. Nevertheless, it remains possible that Major asFS contribute to submicroscopic or low-level mosaicism that is rarely documented in cytogenetic surveys.

## 4. Discussion

In this study, we provide the first systematic cytogenetic characterization of aphidicolin-sensitive fragile sites (asFS) in human pluripotent stem cells. The resulting map offers a valuable reference for interpreting genomic instability events in iPSCs and for targeted screening of recurrent genetic aberrations that arise during culture. The asFS repertoire we identified is tissue-specific and hierarchically organized, comprising a stable universal core and a variable peripheral component. Despite inter-line variability, five loci (9p21, 6q25-26, 20p11-12, 10q22, and Xq25) were consistently fragile across all three lines, accounting for nearly 40% of all breaks at statistically significant sites. This suggests that the fragility of pluripotent cells is not random but reflects a defined, cell-type-specific genomic architecture.

This architecture is further resolved into two hierarchical tiers. The Major cluster, comprising eight highly active loci, accounts for the majority of replication-stress-induced damage despite representing only a quarter of the asFS repertoire. These sites are predominantly universal, linking high activity with cross-line conservation. In contrast, the Minor cluster contains mostly repressed and line-specific loci, which are more susceptible to modulation by epigenetic or stochastic factors.

The functional relevance of Minor asFS is underscored by their preferential co-localisation with breakpoints of recurrent culture-acquired aberrations, including 20q11.21, 1q32, 8q24, 17q21, and 18q21. The asFS that overlap with recurrent aberrations are predominantly Minor sites, whereas the most active Major loci are conspicuously absent from known rearrangement hotspots. This suggests that extreme fragility may produce lesions that are either lethal or lack selective advantage, whereas moderately fragile sites provide viable substrates for clonal dominance.

The integration of MiDAS mapping further refines the landscape by revealing additional replication-stress-sensitive loci in cytogenetically challenging regions (pericentromeric, subtelomeric, and pale G-bands) that are systematically underrepresented in break-based analyses. Novel candidates, such as 16p13, 5p15, 9q32-34, and 7q35-36, illustrate how repair-based mapping can complement cytogenetic break counting, particularly in regions where chromosome morphology limits detection. This dual approach captures both the damage and the repair response, providing a more complete picture of replication-stress sensitivity in iPSCs.

The observation that fragile sites co-localize with both chromosomal gains and losses reflects the mechanistic diversity of replication stress-induced rearrangements. Chromosomal breakage at a fragile site can initiate a spectrum of genomic outcomes depending on the repair pathway engaged and the subsequent selective pressure. Replication stress at large transcribed genes can lead to focal deletions and duplications via fork stalling and template switching (FoSTeS/MMBIR), with deletions concentrated in central, late-replicating regions of transcription units and duplications accumulating at their flanks [45,46,47]. At a larger scale, chromosome arm losses (terminal deletions) can arise from unreplicated DNA entering mitosis, generating acentric lagging chromatin and micronuclei [48]. Under-replicated DNA also gives rise to ultrafine anaphase bridges, which can be resolved through cleavage by nucleases like MUS81-EME1, generating chromatid breaks that may be repaired by NHEJ or MMEJ, frequently yielding deletions and translocations [13]. Alternatively, unresolved UFBs can persist through mitosis, promote chromosome missegregation, and lead to the formation of micronuclei, a well-established source of chromothripsis and other complex rearrangements [46,48]. Furthermore, breakage at fragile sites can initiate breakage-fusion-bridge cycles, driving gene amplification and complex segmental duplications. This is exemplified by the localization of *FRA2C* to the borders of *MYCN* amplicons in neuroblastoma [49]. Sister chromatid exchanges at fragile sites are another manifestation of error-prone repair that can generate both deletions and duplications [13]. Thus, the same underlying fragility can give rise to different classes of aberrations—from focal deletions and duplications to terminal deletions, chromothripsis, and gene amplifications—depending on the repair mechanism engaged and the fitness advantage conferred. The specific outcome—whether a particular rearrangement is retained in the population—is ultimately determined by the selective pressure exerted by culture conditions. This is consistent with our observation that Minor asFS, despite their lower individual activity, show preferential co-localization with breakpoints or MORs of recurrent culture-acquired rearrangements, while the most active Major loci remain conspicuously absent from known rearrangement hotspots. We hypothesize that extreme fragility at Major loci may produce lesions that are either lethal to the cell or confer no selective advantage, whereas moderately fragile Minor sites generate rearrangements that can be tolerated and, when they involve growth-promoting genes, become fixed through clonal selection.

Importantly, many of the identified asFS loci reside within genes associated with neurological functions (e.g., *ANKS1B*, *LINGO2*, *DAB1*, *LSAMP*, *NRXN3*) or with tumor suppressors (*FHIT*, *MACROD2*, *WWOX*, *PARK2*). Although these loci are not themselves recurrently rearranged in iPSC cultures, their potential instability warrants attention, as stochastic events and population bottlenecks during passaging may lead to their involvement in clonal outgrowths. This is particularly relevant for neural differentiation, where the integrity of neuronal genes is critical, and for long-term culture, where loss or amplification of tumor suppressors may affect proliferative potential and genomic stability. Therefore, monitoring the integrity of these loci should be considered when using iPSCs for disease modeling or regenerative medicine applications.

In summary, our findings indicate that the asFS repertoire in iPSCs forms a structured, cell-type-specific map of genomic vulnerability. The distinction between Major and Minor clusters may have functional implications, but we emphasize that the observed co-localization patterns are correlative and do not prove causation. Whether the absence of Major loci from known rearrangement hotspots reflects lethality, a lack of selective advantage, or simply the limits of detection remains an open question. Nevertheless, our work provides a framework for understanding how replication stress and clonal selection shape the mutational landscape of pluripotent stem cells and offers a rationale for further investigation into the relationship between intrinsic fragility and the recurrent karyotypic landscape of cultured iPSCs.

## Data Availability

The original contributions presented in this study are included in the article/Supplementary Material. Further inquiries can be directed to the corresponding author.

## Supplementary Materials

The following supporting information can be downloaded at: https://www.mdpi.com/article/10.3390/cells15171557/s1, Figure S1: Monte Carlo simulation results for threshold determination; Figure S2: Statistical validation of two-cluster solution for 28 fragile sites; Table S1: Identification of aphidicolin-sensitive fragile sites in three iPSC lines; Table S2: Cytogenetic mapping of MiDAS loci in three iPSC lines.

## Abbreviations

The following abbreviations are used in this manuscript:

iPSCs	Induced pluripotent stem cells
asFS	Aphidicolin-sensitive fragile sites
MiDAS	Mitotic DNA synthesis
MORs	Minimal overlapping regions
RS	Replication stress
RFS	Rare fragile site
CFS	Common fragile site
ERFS	Early-replicating fragile site
ISCN	International System for Human Cytogenetic Nomenclature
EdU	5-ethynyl-2′-deoxyuridine
