# Supplementary material for: The Fragile Site Landscape of Induced Pluripotent Stem Cells: Hierarchy, Variability, Tissue Specificity, and Links to Culture-Acquired Rearrangements
Source: Cells. 2026 Aug 28;15(17):1557. doi: 10.3390/cells15171557 (PMC13564719; doi:10.3390/cells15171557)
Supplement: Supplementary file 1 [file cells-15-01557-s001.zip › Suppl Figures S1 and S2.pdf]

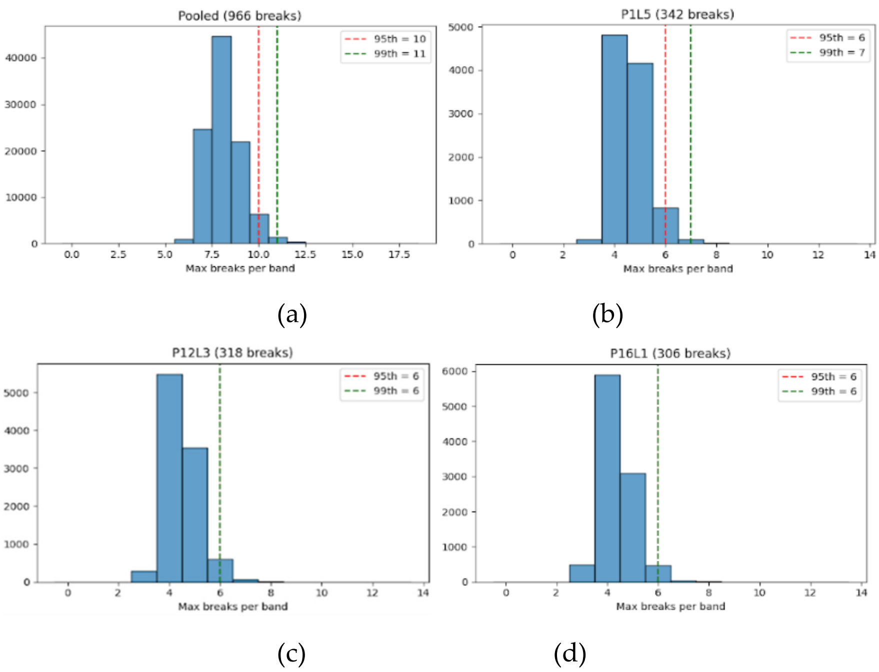

Supplementary Figure S1. Monte Carlo simulation results for threshold determination. Distributions of the maximum number of breaks per band under the null hypothesis of uniform random distribution (100,000 iterations). (**a**) Pooled analysis (966 breaks across 400 bands). The 95th and 99th percentiles are indicated by red and green vertical lines, respectively. The 99th percentile corresponds to 11 breaks; (**b**) Per line analysis for P1L5 (342 breaks). The 99th percentile (7 breaks) is shown as a green line. (**c, d**) Per line analyses for P12L3 (318 breaks) and P16L1 (306 breaks). The 99th percentile (6 breaks) is shown as a green line in each panel.

The histograms demonstrate that under random conditions, the expected maximum rarely exceeds the chosen thresholds, supporting their use for distinguishing genuine fragile sites.

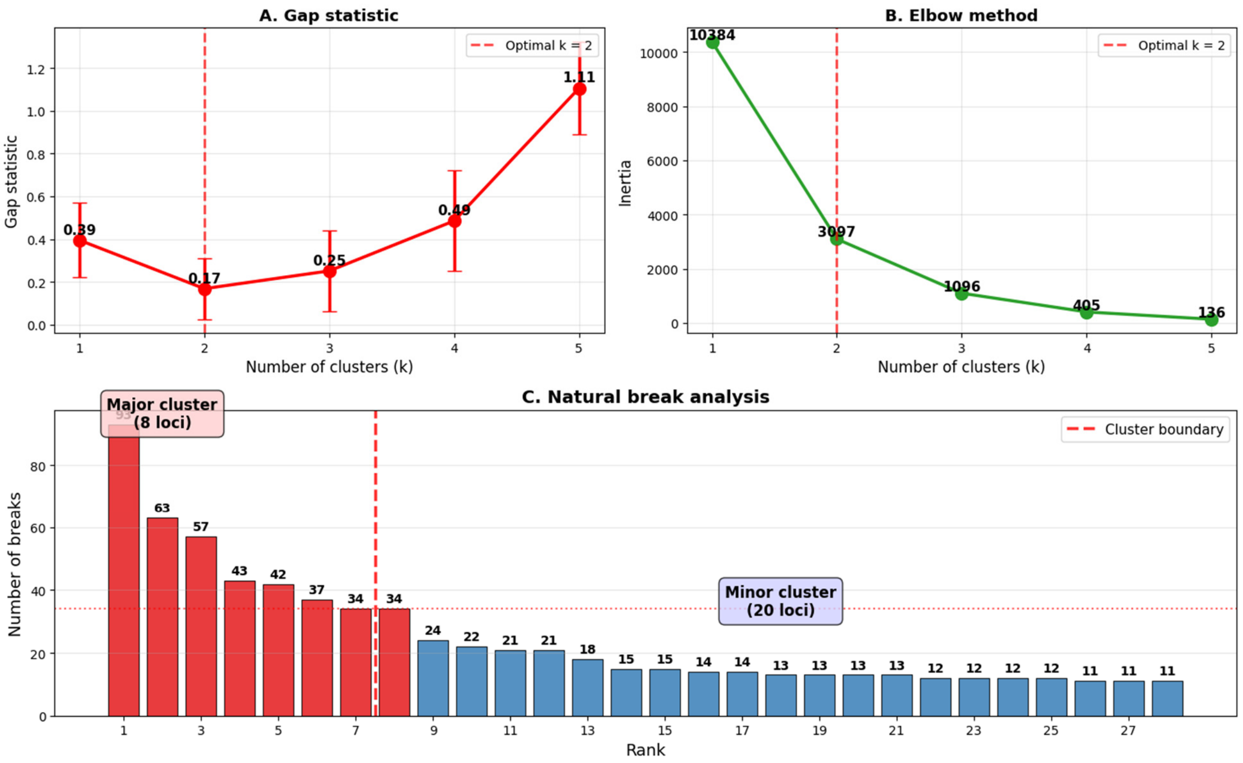

Supplementary Figure S2. Statistical validation of the two-cluster solution for 28 asFS in iPSCs.

(a) Gap statistic. Gap values with standard deviations for k = 1 to 5 clusters. The optimal number of clusters (k = 2) is identified where the gap statistic is maximised relative to its standard error. The red dashed line marks the optimal number of clusters.

(b) Elbow method. Within-cluster inertia as a function of k. A clear inflection point at k = 2 indicates diminishing returns for additional clusters. The red dashed line marks the optimal number of clusters.

(c) Natural break analysis. Ranked break counts for the 28 asFS. A prominent gap separates the 7th locus (34 breaks) from the 8th locus (25 breaks), providing evidence for a natural bipartition of the data. The cluster boundary (red dashed line) is placed after the 7th locus, separating the **Major cluster** (8 loci after X dosage correction, shown in red) from the **Minor cluster** (20 loci, shown in blue). The red dotted horizontal line marks the 34-break threshold.
